# Analytical Model and Experimental Evaluation of the Micro-Scale Thermal Property Sensor for Single-Sided Measurement

**DOI:** 10.3390/mi9040168

**Published:** 2018-04-05

**Authors:** Takashi Katayama, Kaoru Uesugi, Keisuke Morishima

**Affiliations:** Department of Mechanical Engineering, Graduate School of Engineering, Osaka University, 2-1 Yamadaoka, Suita, Osaka 565-0871, Japan; uesugi@live.mech.eng.osaka-u.ac.jp (K.U.); morishima@mech.eng.osaka-u.ac.jp (K.M.)

**Keywords:** thermal property, MEMS sensor, analytical model, single-sided measurement

## Abstract

We report a new analytical model of the MEMS-based thermal property sensor for samples which are difficult to handle and susceptible to damage by thermal stimulus, such as living cells. Many sensor designs had been reported for thermal property measurements, but only a few of them have considered the analytical model of the single-sided measurement in which a measurement sample is placed on the sensor substrate. Even in the few designs that have considered the analytical model, their applicable limits are restricted to more than 1 mm length in practical situations. Our new model considers both the sample and the sensor substrate thermal properties and is applicable to a sensor length less than 1 µm. In order to minimize the influence of the heat stimulus to the sample, the model formulates the required heat dissipating time for different sensor geometries. We propose fast and precise detection circuit architecture to realize our model, and we discuss the sensor performance for a number of different designs.

## 1. Introduction

Researchers in molecular biology have been elucidating nano- to micro-scale mechanisms within about 10 µm diameter spaces that are sustaining life phenomena. The existences of mass transfer, heat generation and temperature response in cells have been explicitly recognized for a long time, and great efforts have been made to define their molecular mechanisms.

Just 9 years after the discovery of the DNA double helix structure, a morphological change of chromosomes induced by temperature stimulation was reported [1]. This discovery led to the discovery of heat shock proteins, and their functions as a molecular chaperone for protein folding were revealed [2,3]. The importance of molecular chaperones in many diseases has been confirmed, and they have become an active research area in recent years [4].

The response of ion channels in a cell membrane to stimuli like temperature was discovered in animal cells [5]. There are different transient receptor potential channels which have different temperature-sensitive ranges of a few degrees, and they act as temperature and other external stimulus sensors [6]. Recently, many efforts are being made in this area, targeting the discovery of analgesic drugs [7].

Two noteworthy features can be seen in these molecular mechanisms relating to the temperature response in living cells. The first is that these mechanisms are seen not in one specific species, but in a wide variety of species. The second is that the responses are not specific for only temperature change, but broadly seen for other stimuli. These features promote an image about the lethality of the excess temperature change inside the cell and the importance of the ability to coexist in the presence of such kinds of external stimuli. The precise observation of heat transfer phenomena in micro spaces such as the intracellular environment has great importance in cell biology.

Thermal property measurements have been recognized as an important research target by not only engineers, but also material scientists [8]. Many methods have been proposed, and non-steady methods are widely used because of their compatibility with accuracy and usability. Non-steady methods quantify the sample thermal properties by generating a temperature change in the sample and measuring its time response. The transient hot wire (THW) method, 3ω method, and laser flash method are typical non-steady methods [9,10,11]. All of these methods model the sample and the heat source geometry, and calculate thermal properties by utilizing the mathematical solution of the heat conduction equation.

MEMS technologies have been widely applied to non-steady methods for the measurement of fluid thermal properties in recent years [12,13,14]. However, MEMS-based non-steady methods have the difficult task of arranging the sample in order to make the setup fit in their theoretical models. Many research studies have been reported based on the free-standing arrangement in which the heat source is separated from the substrate and surrounded mostly by the measurement sample. This arrangement makes it possible to simplify the theoretical model. For example, the THW method derives its theoretical solution by modeling the heat generation from a line heat source and dissipation into a sample in the form of a cylindrical symmetry [15]. Although this arrangement can be easily realized if the sample is a low viscosity fluid, it is difficult or impossible if the sample is a high viscosity fluid or a non-fluid. For our purpose to observe heat transfer phenomena in micro spaces, a single-sided measurement is adequate; in this arrangement the heat source is sandwiched by the measurement sample and sensor substrate. Additionally, by targeting sample properties which are close in value to those of water, it is difficult to ensure a sufficient difference in thermal properties between the sample and sensor substrate. This means we cannot use a semi-infinite model which approximates one of the media as an insulator. Our model should consider both the sample and the substrate thermal properties.

The single-sided measurement can be modeled as two different semi-infinite solids in ideal thermal contact and a heat source on the interface. The analytical solution for the instantaneous point heat source was derived for the 1D problem by Sommerfelt [16] in 1894, and for the 3D problem by Bellman, et al. [17] in 1949. The latter is referred to here as the B.M.W. solution. Gustafsson [18] and Birge [19] realized the single-sided measurement by employing the 1D model which was based on the approximation that the heat source extends infinitely. This approximation is valid when the measurement is done in a very short time in which most of the heat can be assumed to spread only in the vertical direction of the finite size of the heat source in practical situations. This requirement can be formulated as below [18]:

(1) $$\frac{\sqrt{\alpha t}}{X}\ll \sqrt{4\pi }$$

where α is the thermal diffusivity, t is the heating time and X is the characteristic length. Equation (1) gives the limitation on the sample miniaturization. Moreover, in the 1D model, because the thermal properties appear in the form of $k^{2}/\alpha$, they cannot be acquired separately (k is the thermal conductivity).

By constructing the model from the B.M.W. solution, these difficulties can be avoided. However, because of its complex equation, few examples exist which apply the B.M.W. solution to practical problems [20]. A series of papers by Shendeleva [21,22], and our previous work [23] on the characteristics of the time evolution of the B.M.W. solution make it possible to have a physical interpretation of this solution.

In this paper, we construct the analytical heat conduction model for the MEMS-based single-sided measurement sensor from the B.M.W. solution. By loosening the infinite approximation which is used in the conventional models, we establish the new model which can be applied to µm scale samples. We also discuss the measurement conditions which can minimize the influence of the temperature elevation of the sample. A metal film electrode on the substrate works as both the heat source and the temperature detector. A fast and precise electrical circuit is implemented and the basic characteristics of sensors having different dimensions are discussed.

## 2. Principle

### 2.1. The B.M.W. Solution for the Condition That the Heat Source Is on the Interface

**Figure 1:**
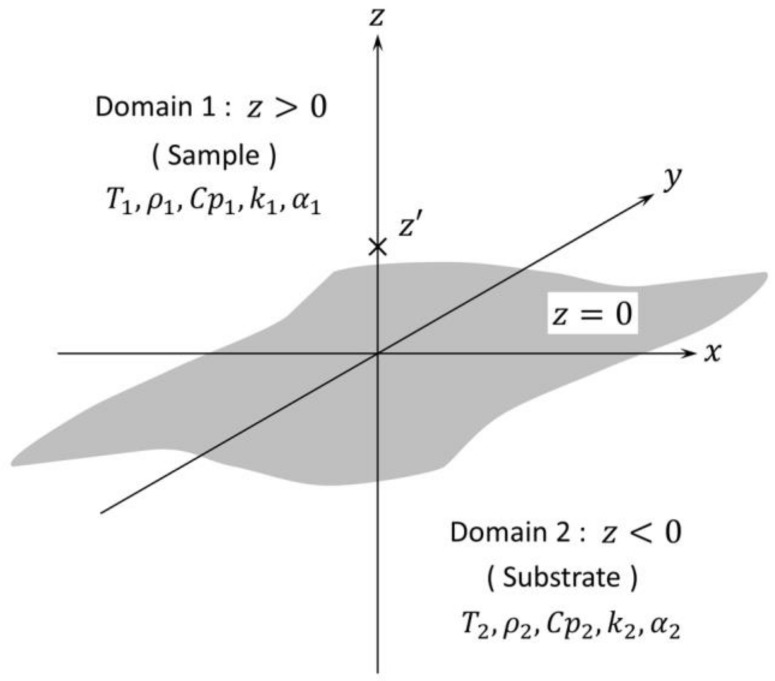
Model for the B.M.W. solution. T (unit: K), temperature; ρ (unit: kg/m^3^), density; Cp (unit: J/(kg × K)), specific heat capacity; k (unit: W/(m × K)), thermal conductivity; α (unit: m^2^/s), thermal diffusivity; $z^{\prime }>0$, position of the point heat source. The subscript designates its domain.

The basic model by Bellman et al. [17] is described in Figure 1. Two semi-infinite domains which have different thermal properties are bordering on z=0 in ideal thermal contact and zero initial temperature. In our research, domain 1 is assumed as the sample and domain 2 is assumed as the sensor substrate. The instantaneous point heat source is arranged at $\left(x,y,z\right)=\left(0,0,z^{\prime }\right),\;\left(z^{\prime }>0\right)$, and it generates q (unit: J) heat. Both domains have different thermal properties, but are assumed to be isotropic, uniform and constant. We ignore convection and radiation. In our research, the metal electrode which acts as the heat source is placed on the interface, then $z^{\prime }\to 0$. The metal electrode is thin enough that its thickness can be assumed to be zero.

The solution can be written as follows.

(2) $$\underset{z^{\prime }\to 0}{\lim}T_{1}\left(x,y,z,z^{\prime },t\right)=\frac{q}{\rho_{1}Cp_{1}}\times \frac{\beta^{2}}{8\pi^{2}{\left(\alpha_{1}t\right)}^{\frac{3}{2}}}\int_{0}^{1}\frac{\exp\left\{-\frac{\beta^{2}R^{2}}{4\left(\beta^{2}u+1-u\right)}\right\}}{\left(\beta^{2}u+1-u\right)\sqrt{u\left(1-u\right)}}f_{1}\left(Z,\sigma ,u\right)du,$$

(3) $$f_{1}\left(Z,\sigma ,u\right)=\frac{-\sigma^{3}uZ}{{\left(\sigma^{2}u+1-u\right)}^{2}}\exp\left(-\frac{Z^{2}}{4u}\right)\;+\frac{\sigma \sqrt{\pi u\left(1-u\right)}}{{\left(\sigma^{2}u+1-u\right)}^{\frac{3}{2}}}\left\{1-\frac{Z^{2}\sigma^{2}}{2\left(\sigma^{2}u+1-u\right)}\right\}\exp\left\{-\frac{Z^{2}\sigma^{2}}{4\left(\sigma^{2}u+1-u\right)}\right\}\mathrm{erfc}\left\{\frac{-Z\left(1-u\right)}{2\sqrt{u\left(1-u\right)\left(\sigma^{2}u+1-u\right)}}\right\},$$

(4) $$\underset{z^{\prime }\to 0}{\lim}T_{2}\left(x,y,z,z^{\prime },t\right)=\frac{q}{\rho_{1}Cp_{1}}\times \frac{\beta^{2}}{8\pi^{2}{\left(\alpha_{1}t\right)}^{\frac{3}{2}}}\int_{0}^{1}\frac{\exp\left\{-\frac{\beta^{2}R^{2}}{4\left(\beta^{2}u+1-u\right)}\right\}}{\left(\beta^{2}u+1-u\right)\sqrt{u\left(1-u\right)}}f_{2}\left(Z,\sigma ,u\right)du,$$

(5) $$f_{2}\left(Z,\sigma ,u\right)=\frac{\beta Z\left(1-u\right)}{{\left(\sigma^{2}u+1-u\right)}^{2}}\exp\left\{-\frac{\beta^{2}Z^{2}}{4\left(1-u\right)}\right\}\;+\frac{\sigma \sqrt{\pi u\left(1-u\right)}}{{\left(\sigma^{2}u+1-u\right)}^{\frac{3}{2}}}\left\{1-\frac{{\left(\beta Z\right)}^{2}}{2\left(\sigma^{2}u+1-u\right)}\right\}\exp\left\{-\frac{{\left(\beta Z\right)}^{2}}{4\left(\sigma^{2}u+1-u\right)}\right\}\mathrm{erfc}\left\{\frac{\sigma \beta Zu}{2\sqrt{u\left(1-u\right)\left(\sigma^{2}u+1-u\right)}}\right\},$$

(6) $$r^{2}=x^{2}+y^{2},\;R=\frac{r}{\sqrt{\alpha_{1}t}},\;Z=-\frac{z}{\sqrt{\alpha_{1}t}},\;\beta =\frac{\sqrt{\alpha_{1}}}{\sqrt{\alpha_{2}}},\;\sigma =\frac{k_{2}\sqrt{\alpha_{1}}}{k_{1}\sqrt{\alpha_{2}}}.$$

We are interested in the temperature on the interface. Then z→0 can be used to simplify Equations (2)–(5) to (7).

(7) $$\begin{array}{l} T_{p}\left(x,y,t\right)=\underset{z,z^{\prime }\to 0}{\lim}T_{1}\left(x,y,z,z^{\prime },t\right)=\underset{z,z^{\prime }\to 0}{\lim}T_{2}\left(x,y,z,z^{\prime },t\right)\\ \;=\frac{q}{\rho_{1}Cp_{1}}\times \frac{\sigma \beta^{2}}{8{\left(\pi \alpha_{1}t\right)}^{\frac{3}{2}}}\int_{0}^{1}\frac{\exp\left\{-\frac{\beta^{2}R^{2}}{4\left(\beta^{2}u+1-u\right)}\right\}}{\left(\beta^{2}u+1-u\right){\left(\sigma^{2}u+1-u\right)}^{\frac{3}{2}}}du. \end{array}$$

### 2.2. The Continuous Rectangle Heat Source Solution on the Interface of Two Semi-Infinite Domains

**Figure 2:**
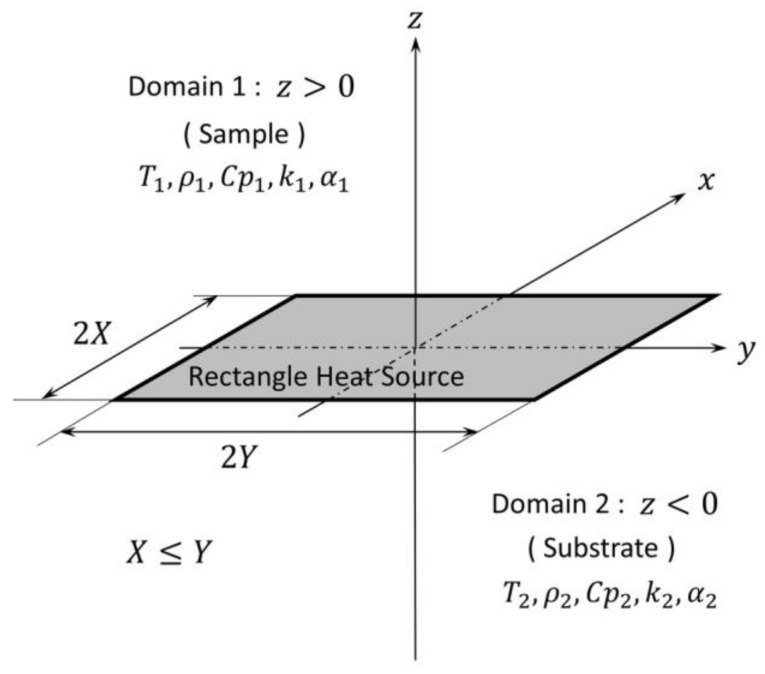
Model for the rectangle heat source on the interface of two semi-infinite domains.

The model of our problem is described in Figure 2. We discuss the average temperature of the continuous rectangle heat source on the interface whose short side length is 2X and long side length is 2Y. This kind of problem can be derived by calculating the time and spatial integral of the instantaneous point heat source solution $T_{p}$.

(8) $$\bar{T_{r}}\left(t\right)=\frac{1}{4XY}\int_{-Y}^{Y}dy\int_{-X}^{X}dx\int_{0}^{t}dt^{\prime }\int_{-Y}^{Y}dy^{\prime }\int_{-X}^{X}dx^{\prime }T_{p}\left(x-x^{\prime },y-y^{\prime },t-t^{\prime }\right)$$

The heat source thickness $Z_{t}$ is assumed to be zero.

The injected heat q (unit: J) in Equations (2) and (4) is now the injected heat $p\;\left(\mathrm{unit}:\;J/\left(m^{2}\times s\right)\right)\;$ for the unit area and the unit time in Equation (8). Equation (8) is complex because there are sextuple integrals including the integral by u in $T_{p}$. However, because the integrand is integrable, the order of the integral is interchangeable by Fubini’s theorem, so the integrals except for u can be reduced to Equation (9).

(9) $$I\left(t\right)=\int_{0}^{t}dt^{\prime }\int_{-Y}^{Y}dy\int_{-X}^{X}dx\int_{-Y}^{Y}dy^{\prime }\int_{-X}^{X}dx^{\prime }\frac{1}{{\left\{4\pi \alpha_{1}\left(t-t^{\prime }\right)\right\}}^{\frac{3}{2}}}\exp\left[-\frac{\left\{{\left(x-x^{\prime }\right)}^{2}+{\left(y-y^{\prime }\right)}^{2}\right\}}{4\alpha_{1}\left(t-t^{\prime }\right)}\right]$$

The integrals of $x,x^{\prime }$ and $y,y^{\prime }$ can be treated separately.

(10) $$\begin{array}{l} I\left(t\right)=\int_{0}^{t}dt^{\prime }\frac{1}{\sqrt{4\alpha_{1}\pi \left(t-t^{\prime }\right)}}\times \left[\frac{\sqrt{4\alpha_{1}\left(t-t^{\prime }\right)}}{\sqrt{\pi }}\exp\left\{-\frac{X^{2}}{\alpha_{1}\left(t-t^{\prime }\right)}\right\}-\frac{\sqrt{4\alpha_{1}\left(t-t^{\prime }\right)}}{\sqrt{\pi }}+2X\mathrm{erf}\left(\frac{X}{\sqrt{\alpha_{1}\left(t-t\prime \right)}}\right)\;\right]\\ \;\times \left[\frac{\sqrt{4\alpha_{1}\left(t-t^{\prime }\right)}}{\sqrt{\pi }}\exp\left\{-\frac{Y^{2}}{\alpha_{1}\left(t-t^{\prime }\right)}\right\}-\frac{\sqrt{4\alpha_{1}\left(t-t^{\prime }\right)}}{\sqrt{\pi }}+2Y\mathrm{erf}\left(\frac{Y}{\sqrt{\alpha_{1}\left(t-t^{\prime }\right)}}\right)\;\right] \end{array}$$

The terms of exp×erf and erf×erf cannot be integrated into the closed-form solutions.

The model of the continuous line heat source in the infinite uniform media, which is adopted by the THW method, assumes $0\leftarrow Z_{t}=X\ll Y\to \infty$ [15]. X→0 allows the avoidance of integrations of x and $x^{\prime }$, and Y→∞ makes the integrals of y and $y^{\prime }$ unity. By these considerations, the THW method successfully extracts the thermal conductivity from the temperature transient response.

The transient hot strip (THS) method, which is also a non-steady method to measure thermal properties, models the continuous strip heat source in the infinite uniform media [24,25]. The THS method assumes $0\leftarrow Z_{t}\ll X\ll Y\to \infty$, and the integrations of x and $x^{\prime }$ are treated. As a merit of this consideration, the THS method successfully extracts the thermal conductivity and the thermal diffusivity, independently. And also, the non-zero 2X gives the averaging function towards the x direction. This feature provides an advantage to the THS method in non-fluid sample measurements which cannot be assumed to be uniform.

Advances in microfabrication technologies permit a decrease for practical $Z_{t}$ values close to the physical limit $\approx 1\times 10^{-10}\;m$. If the targeted sample size is $\phi 10\;\mu m=1\times 10^{-5}\;m$ or less, the requirement of Y→∞ must be eliminated and the integrations of Equation (10) must be treated.

We focus on the right-most term of Equation (10).

(11) $$\begin{array}{c} \frac{\sqrt{4\alpha_{1}\left(t-t^{\prime }\right)}}{\sqrt{\pi }}\exp\left\{-\frac{Y^{2}}{\alpha_{1}\left(t-t^{\prime }\right)}\right\}-\frac{\sqrt{4\alpha_{1}\left(t-t^{\prime }\right)}}{\sqrt{\pi }}+2Y\mathrm{erf}\left(\frac{Y}{\sqrt{\alpha_{1}\left(t-t^{\prime }\right)}}\right)\\ =2Y\left[\frac{1}{\sqrt{\pi }\Phi }\left\{\exp\left(-\Phi^{2}\right)-1\right\}+\mathrm{erf}\left(\Phi \right)\right]=2Y\times F\left(\Phi \right)\\ \;\Phi =\frac{Y}{\sqrt{\alpha_{1}\left(t-t^{\prime }\right)}} \end{array}$$

**Figure 3:**
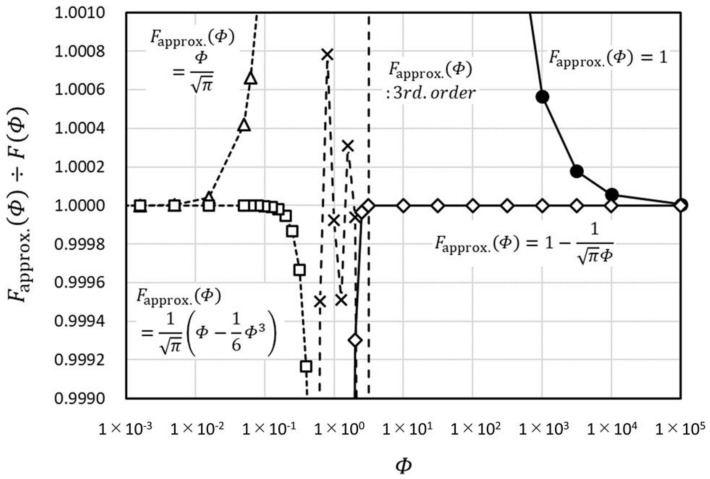
Comparison of the relative approximation error with various approximation functions.

We consider the approximation of F as $F_{\mathrm{approx}.}$. The relative approximation error for several $F_{\mathrm{approx}.}$ which can be used in different ranges of Φ are depicted in Figure 3.

$F_{\mathrm{approx}.}=1$ is equivalent to Y→∞ in the THW and THS methods. By taking $F_{\mathrm{approx}.}=1-1/\left(\Phi \sqrt{\pi }\right)$, we can widen the applicable range of Y $10^{-4}\;\mathrm{to}\;10^{-3}$ times to the lower side.

In non-steady methods, the dimensionless parameter constructed from spatial variables, a time variable, and thermal diffusivity, has important roles in the measurement conditions as can been seen from Equations (1) and (11). When miniaturization of the sample is required, miniaturization of the heat source, which means shrinkage of the spatial variables, is required. On the other hand, the heating time defines the heat diffusion distance from the heat source. If the time variable is not reduced along with the spatial variables, a widespread temperature field around the heat source will be created. The widespread temperature field is affected by the properties of large regions, then the effect of the miniaturization of the heat source is compensated.

As we discuss in the Materials and Methods section, 1 µV or 10^−6^ order of the precise measurement is required by non-steady methods using the resistance temperature detector. Generally speaking, precision and speed are difficult to achieve together in the detection circuitry. To achieve 10^−6^ order precision, there is a practical limitation in speed around $10^{7}\;\mathrm{Hz}$ or $10^{-7}\;s$. $Y_{\min}$, the minimum value of Y, is estimated as about $1.2\;\times \;10^{-3}\;m$, if we use $F_{\mathrm{approx}.}=1\;\left(\Phi_{\min}\approx 1\times 10^{4}\right)$, $∆t=1\times 10^{-7}\;s$, and water as the sample $\left(\alpha_{1}=1.47\times 10^{-7}\;m^{2}/s\right)$. If we use $F_{\mathrm{approx}.}=1-1/\left(\Phi \sqrt{\pi }\right)$, then $Y_{\min}\approx 3.0\times 10^{-7}\;m$ and this can satisfy our targeted sample size.

By using the approximation of $F_{\mathrm{approx}.}=1-1/\left(\Phi \sqrt{\pi }\right)$, we can get the closed form solution for the integration of Equation (10).

(12) $$\begin{array}{l} I\left(t\right)\approx \int_{0}^{t}dt\prime \frac{1}{\sqrt{4\alpha_{1}\pi \left(t-t\prime \right)}}\times \left[\frac{\sqrt{4\alpha_{1}\left(t-t\prime \right)}}{\sqrt{\pi }}\exp\left\{-\frac{X^{2}}{\alpha_{1}\left(t-t\prime \right)}\right\}-\frac{\sqrt{4\alpha_{1}\left(t-t\prime \right)}}{\sqrt{\pi }}+2X\mathrm{erf}\left(\frac{X}{\sqrt{\alpha_{1}\left(t-t\prime \right)}}\right)\;\right]\\ \;\times \left\{2Y-\frac{\sqrt{4\alpha_{1}\left(t-t\prime \right)}}{\sqrt{\pi }}\right\}\\ =2Y\times \frac{2X^{2}}{\alpha_{1}\sqrt{\pi }}[\frac{\sqrt{\alpha_{1}t\;}}{X}\left\{1-\frac{1}{3\sqrt{\pi }Y}\left(\frac{X^{2}}{\sqrt{\alpha_{1}t}}+\frac{3\sqrt{\alpha_{1}t\;}}{2}\right)\right\}\mathrm{erf}\left(\frac{X}{\sqrt{\alpha_{1}t}}\right)+\frac{1}{3\sqrt{\pi }Y}\left\{X+\frac{{\left(\alpha_{1}t\right)}^{\frac{3}{2}}}{\sqrt{\pi }\;X^{2}}\right\}\\ \;-\frac{\alpha_{1}t}{X^{2}\sqrt{4\pi }}-\left\{\frac{1}{3\sqrt{\pi }Y}\left\{\frac{\sqrt{\alpha_{1}t}}{\sqrt{\pi }}+\frac{{\left(\alpha_{1}t\right)}^{\frac{3}{2}}}{\sqrt{\pi }X^{2}}\right\}-\frac{\alpha_{1}t}{X^{2}\sqrt{4\pi }}\right\}\exp\left(-\frac{X^{2}}{\alpha_{1}t}\right)-\frac{1}{\sqrt{4\pi }\;}\mathrm{Ei}\left(-\frac{X^{2}}{\alpha_{1}t}\right)] \end{array}$$

I(t)/(2Y) will be converged to the solution of the THS method in the limit of Y→∞. By utilizing Equations (12) to (8), we can get the solution of our model.

(13) $$\begin{array}{c} \bar{T_{r}}\left(t\right)=\frac{p}{\rho_{1}Cp_{1}}\times \int_{0}^{1}du\frac{X}{\sqrt{\alpha_{1}\alpha^{*}}\times \sqrt{\pi }}\times \frac{\sigma }{{\left(\sigma^{2}u+1-u\right)}^{\frac{3}{2}}}\times f^{*}\\ f^{*}=\frac{\sqrt{\alpha^{*}t}}{X}\left\{1-\frac{1}{3\sqrt{\pi }Y}\left(\frac{X^{2}}{\sqrt{\alpha^{*}t}}+\frac{3\sqrt{\alpha^{*}t}}{2}\right)\right\}\mathrm{erf}\left(\frac{X}{\sqrt{\alpha^{*}t}}\right)+\frac{1}{3\sqrt{\pi }Y}\left\{X+\frac{{\left(\alpha^{*}t\right)}^{\frac{3}{2}}}{\sqrt{\pi }X^{2}}\right\}-\frac{\alpha^{*}t}{2\sqrt{\pi }X^{2}}\\ \;-\left\{\frac{1}{3\sqrt{\pi }Y}\left(\frac{\sqrt{\alpha^{*}t}}{\sqrt{\pi }}+\frac{{\left(\alpha^{*}t\right)}^{\frac{3}{2}}}{\sqrt{\pi }X^{2}}\right)-\frac{\alpha^{*}t}{\sqrt{4\pi }X^{2}}\right\}\exp\left(-\frac{X^{2}}{\alpha^{*}t}\right)-\frac{1}{\sqrt{4\pi }}\mathrm{Ei}\left(-\frac{X^{2}}{\alpha^{*}t}\right)\\ \alpha^{*}=\frac{\left(\beta^{2}u+1-u\right)}{\beta^{2}}\alpha_{1} \end{array}$$

By comparing Equation (13) to the solution of the THS method $\bar{T_{s}}\left(t\right)$, we can follow the consideration in the THS method [25]:

(14) $$\bar{T_{s}}\left(t\right)=\frac{p}{\rho Cp}\times \frac{X}{\alpha \sqrt{\pi }}\times f,\;f=\frac{\sqrt{\alpha t}}{X}\mathrm{erf}\left(\frac{X}{\sqrt{\alpha t}}\right)-\frac{\alpha t}{\sqrt{4\pi }X^{2}}\left\{1-\exp\left(-\frac{X^{2}}{\alpha t}\right)\right\}-\frac{1}{\sqrt{4\pi }}\mathrm{Ei}\left(-\frac{X^{2}}{\alpha t}\right),$$

where f is the dimensionless function and all of the time variables are stored in f. The shape of the time–temperature function is basically defined by f. α is the solo physical parameter contained in f. On the other hand, there is the term 1/(ρCpα) outside f, and this is equivalent to the reciprocal of the thermal conductivity 1/k. The THS method utilizes these features to extract the thermal conductivity and thermal diffusivity, independently.

The similar consideration can be applied to Equation (13). $f^{*}$ is also a dimensionless function just as f is, and all of the time variables are stored in $f^{*}$. There are Y variables in $f^{*}$ in contrast to f in which we take the limit of Y→∞. $\alpha^{*}$ is the only physical parameter contained in $f^{*}$. As we already reported elsewhere [23], $\alpha^{*}$ has a meaning that it is the physical parameter linearly scanned from $\alpha_{2}$ to $\alpha_{1}$ through the integration by the dimensionless parameter u. Thus, $f^{*}$ also defines the shape of the time–temperature function, and the thermal diffusivity is the solo physical parameter. $\alpha^{*}$ is constructed by two thermal diffusivity parameters $\alpha_{1}$ and $\alpha_{2}$. The thermal diffusivity of the sensor substrate $\alpha_{2}$ can be assumed to be a fixed value during an experiment. As a result, the shape of the time–temperature function is defined solely by the sample thermal diffusivity $\alpha_{1}$.

The outside term of $f^{*}$ is complex but has similar physical meanings to the outside term of f.

(15) $$1/\left(\rho_{1}Cp_{1}\times \sqrt{\alpha_{1}\alpha^{*}}\right)=\frac{1}{k_{1}}\times \sqrt{\frac{\alpha_{1}}{\alpha^{*}}},$$

Because $\alpha^{*}$ and $\alpha_{1}$ can be gotten from $f^{*}$, we can extract $k_{1}$ and $\alpha_{1}$ independently from Equation (13).

### 2.3. Dissipation of the Injected Heat

Non-steady methods generally heat the sample and calculate the thermal properties from the time–temperature response. To achieve the small size sample measurement, not only miniaturization of the heat source, but also faster measurement is required. Faster measurement leads to the decrease of the S/N ratio of the detection circuit. In order to compensate for the decrease of the S/N ratio, the increase of the injected heat, taking the average of multiple heating events or their combination is generally adopted.

On the other hand, any one of these three countermeasures easily leads to the increase of the sample temperature. To minimize the increase of the sample temperature, a sufficient waiting period between multiple heating events must be ensured. In this subsection, we consider the dissipation time of the injected heat, depending on the heat source geometry.

The temperature-decreasing ratio during the waiting period for the instantaneous arbitrary geometry heat source can be written as below.

(16) $$\frac{T_{c}}{T_{h}}=\frac{\int_{0}^{t_{h}}T_{i}\left(t_{c}-t^{\prime }\right)dt^{\prime }}{\int_{0}^{t_{h}}T_{i}\left(t_{h}-t^{\prime }\right)dt^{\prime }},$$

$T_{h}$ is the temperature just after $t_{h}$ time period heating and $T_{c}$ is the temperature after $t_{c}-t_{h}$ time period waiting from the end of $t_{h}$ time period heating. $T_{i}\left(t\right)$ is the temperature of the instantaneous arbitrary geometry heat source at time t. $T_{i}\left(t\right)$ takes different values depending on the heat source geometry, and $T_{c}/T_{h}$ also depends on the geometry. The temperature decreasing ratios at r distance from the heat source for the geometries, that is, point, line, and plane are as written below.

(17) $${\left(\frac{T_{c}}{T_{h}}\right)}_{\mathrm{point}}=\frac{\mathrm{erf}\left\{\frac{1}{\sqrt{\left(c-1\right)}}V\right\}-\mathrm{erf}\left(\frac{1}{\sqrt{c}}V\right)}{1-\mathrm{erf}\left(V\right)}$$

(18) $${\left(\frac{T_{c}}{T_{h}}\right)}_{\mathrm{line}}=\frac{\mathrm{Ei}\left\{-\frac{V^{2}}{\left(c-1\right)}\right\}-\mathrm{Ei}\left(-\frac{V^{2}}{c}\right)}{-\mathrm{Ei}\left(-V^{2}\right)}$$

(19) $${\left(\frac{T_{c}}{T_{h}}\right)}_{\mathrm{plane}}=\frac{-\frac{\sqrt{c-1}}{\sqrt{\pi }}\exp\left\{-\frac{V^{2}}{\left(c-1\right)}\right\}-V\mathrm{erf}\left\{\frac{V}{\sqrt{\left(c-1\right)}}\right\}+\frac{\sqrt{c}}{\sqrt{\pi }}\exp\left(-\frac{V^{2}}{c}\right)+V\mathrm{erf}\left(\frac{V}{\sqrt{c}}\right)}{\frac{1}{\sqrt{\pi }}\exp\left(-V^{2}\right)-V\mathrm{erfc}\left(V\right)}$$

**Figure 4:**
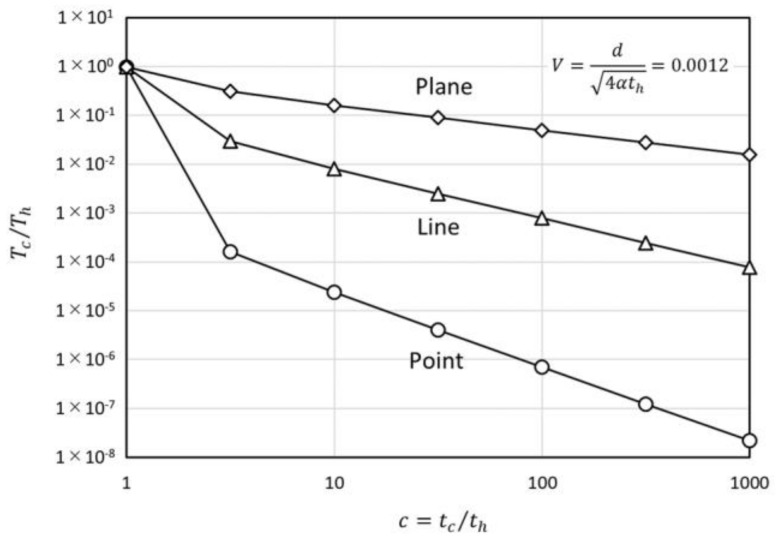
Temperature-decreasing ratio of the different heat source geometries, point, line, and plane.

Here, $V=r/\sqrt{4\alpha t_{h}}$ and $c=t_{c}/t_{h}$. Equations (17)–(19) are depicted in Figure 4. The gradients of the decreasing ratios are along $t^{-3/2},\;t^{-2/2},\;t^{-1/2}$ for point, line and plane heat source geometries, respectively. These features are easily read from each instantaneous heat source solution [26]. From a physical viewpoint, the heat can dissipate three-dimensionally from the point source, two-dimensionally from the line source, and one-dimensionally from the plane source.

**Figure 5:**
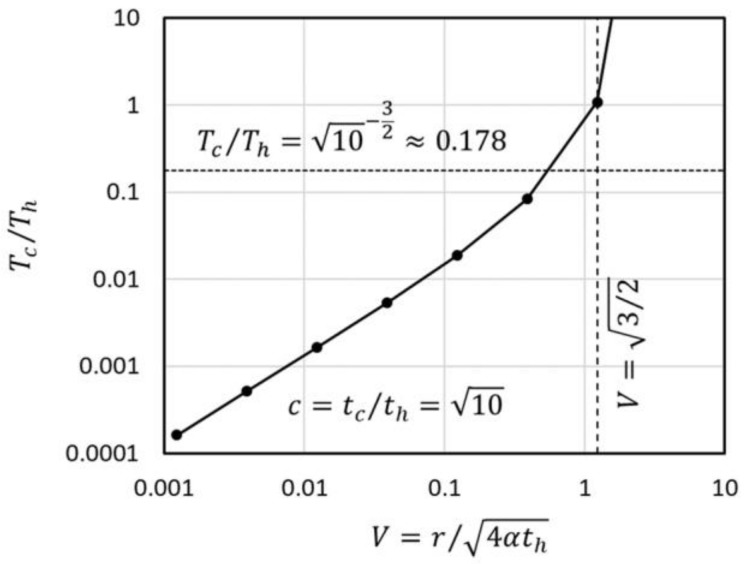
Temperature-decreasing ratio of the point heat source immediately after the end of heating.

The gradient in the time region just after the end of heating (c≈1) depends on V. The decreasing ratio at $c=\sqrt{10}$ for the point heat source is depicted in Figure 5. In the region of $V>\sqrt{3/2}$, the decreasing ratio exceeds unity because the temperature peak induced by the heat injection does not reach the observation position. In the small V region, the ratio takes the value less than ${\left(\sqrt{10}\right)}^{-3/2}$ which corresponds to the $t^{-3/2}$ rule. This is because the non-linearity of the decreasing ratio in the small t region is relatively large. This tendency exists in any source geometry, but it is strongest in the point heat source geometry because of its having the largest non-linearity.

The temperature-decreasing ratio for the THS method in which the strip heat source is used can be as described below:

(20) $$\begin{array}{l} {\left(\frac{\bar{T_{c}}}{\bar{T_{h}}}\right)}_{\mathrm{strip}}=[4P\sqrt{\pi }\left\{\sqrt{c}\mathrm{erf}\left(\frac{2P}{\sqrt{c}}\right)-\sqrt{\left(c-1\right)}\mathrm{erf}\left\{\frac{2P}{\sqrt{\left(c-1\right)}}\right\}\right\}\\ \;-4P^{2}\left\{\mathrm{Ei}\left(-\frac{4P^{2}}{c}\right)-\mathrm{Ei}\left\{-\frac{4P^{2}}{\left(c-1\right)}\right\}\right\}+\left\{c\exp\left(-\frac{4P^{2}}{c}\right)-\left(c-1\right)\exp\left\{-\frac{4P^{2}}{\left(c-1\right)}\right\}\right\}-1]\\ \;÷\left[4\sqrt{\pi }P\mathrm{erf}\left(2P\right)-4P^{2}\mathrm{Ei}\left(-4P^{2}\right)+\exp\left(-4P^{2}\right)-1\right],\\ \;\mathrm{where}\;P=X/\sqrt{4\alpha t_{h}} \end{array}$$

**Figure 6:**
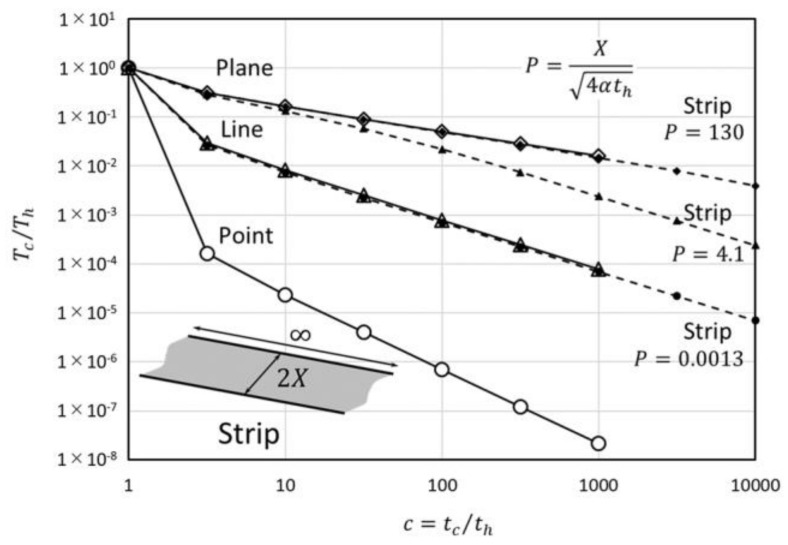
Temperature-decreasing ratio of the strip heat source.

In Equation (20), the average temperature of the strip is considered. Equation (20) is depicted in Figure 6 by comparison with point, line and plane heat source geometries. When P takes a small value, it means a narrower strip, and the gradient of the decreasing ratio is similar to the gradient of the line source $t^{-2/2}$. When P takes a larger value, it means a wider strip, and the gradient is similar to the gradient of the plane source $t^{-1/2}$ at first, but then goes down to $t^{-2/2}$. On the other hand, because the length of the strip is infinite, the heat flow to the length direction will not rise and the gradient will not exceed $t^{-2/2}$.

The temperature-decreasing ratio for the rectangle heat source is as described by Equation (21):

(21) $$\begin{array}{l} {\left(\frac{\bar{T_{c}}}{\bar{T_{h}}}\right)}_{\mathrm{rect}}\approx \left[\frac{2P}{\pi W}\left\{\frac{1}{\sqrt{\left(c-1\right)}}-\frac{1}{\sqrt{c}}\right\}\right]\\ \;÷[\frac{1}{2P}\left\{1-\frac{W}{3\sqrt{\pi }}\left(2P+\frac{3}{4P}\right)\right\}\mathrm{erf}\left(2P\right)+\frac{W}{3\sqrt{\pi }}\left\{1+\frac{1}{8\sqrt{\pi }P^{3}}\right\}\\ \;-\frac{1}{8P^{2}\sqrt{\pi }}-\left\{\frac{W}{3\sqrt{\pi }}\left(\frac{1}{2P\sqrt{\pi }}+\frac{1}{8\sqrt{\pi }P^{3}}\right)-\frac{1}{8P^{2}\sqrt{\pi }}\right\}\exp\left(-4P^{2}\right)-\frac{1}{\sqrt{4\pi }\;}\mathrm{Ei}\left(-4P^{2}\right)],\\ \;\mathrm{where}\;P=\frac{X}{\sqrt{4\alpha t_{h}}},\;\mathrm{and}\;W=\frac{X}{Y}. \end{array}$$

**Figure 7:**
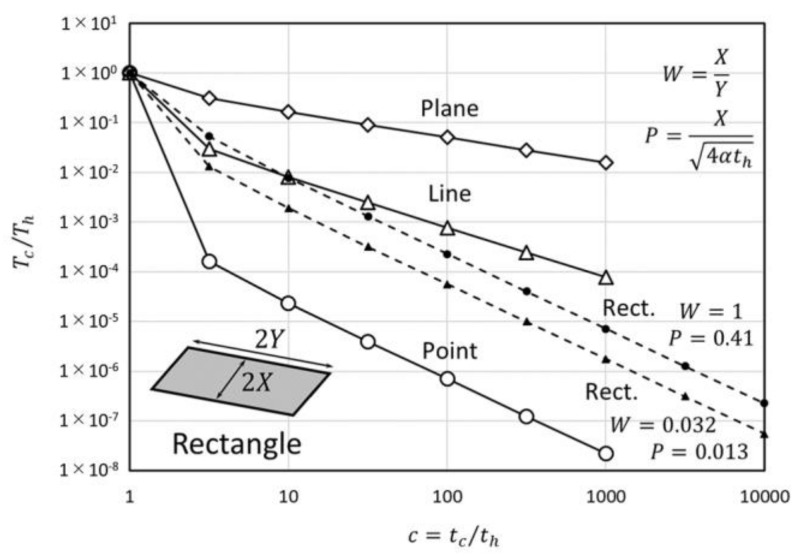
Temperature-decreasing ratio of the rectangle heat source.

In Equation (21), the average temperature on the rectangle heat source is considered to be the same as for Equation (20). In addition, the approximation of $F_{\mathrm{approx}.}=\Phi /\sqrt{\pi }$ which can be used in the large $t_{c}$ region is applied to calculate $\bar{T_{c}}$ for both directions of x and y in Equation (21). Equation (21) is depicted in Figure 7 by comparison with point, line and plane heat source geometries. The gradient of the decreasing ratio is $t^{-3/2}$ and it is similar to the gradient for the point source. Because the heat can dissipate to the length direction, the temperature decreases faster than for the strip source. The shortening of the heat source length has a merit for thermally fragile samples because of its fast heat dissipation feature.

## 3. Materials and Methods

In this section, we propose a practical setup to realize the conditions described in the theoretical Equation (13). In addition, we discuss the electrical circuit which can measure temperature change precisely and minimize the temperature elevation in the sample.

### 3.1. General Description

We form the metal rectangle pattern on the substrate and use it as both the heat source and the temperature detector. This configuration allows both measurement precision and ease of sensor-sample arrangement which has compatibility to our theoretical model. The injection of heat can be achieved by the introduction of a precisely quantified electrical current to the rectangle pattern. The measurement of the time–temperature response is carried out by utilizing the temperature coefficient of the resistance (TCR) of the metal. By measuring the change of the voltage drop in the rectangle pattern during the introduction of the electrical current, we can get information about the temperature change on the rectangle pattern. The geometries of the heating area and the temperature measuring area are identical and this is one of the reasons why this configuration offers high precision.

On the other hand, the TCR value of nickel which is well known as a high TCR material, is only about $7\times 10^{-3}\;K^{-1}$ [27]. The elevation of the temperature should be limited to the order of ∆1 K, so in order to realize precise analysis, the required resolution should be around $10^{-3}\;K$. This means we have to treat a 1 ppm order voltage change. As discussed in Section 2.2, if we target water as a sample, and use a 1 µm sample size, we have to complete the measurement within 1 µs. To meet the opposing requirements, achieving both precision and speed is the challenge of this method.

A common countermeasure which is adopted by existing methods like the 3ω method [28] and the pulse THS method [29] is repeating measurements. These methods do not rely on a solo time-temperature event; rather they use multiple heating events and ensure precision by taking the average of a number of time–temperature responses. In addition, in these existing methods, in order to avoid the problem of the measurement dynamic range, the voltage change which does not relate to the temperature change is canceled out by means of an ingenious circuitry architecture like the bridge circuit.

We divide the problem into the aspects of “Precision” and “Trueness” [30]. Precision must be ensured for our purpose, and averaging does this effectively. In contrast, trueness is not so important and the extra complex circuitry can be a disadvantage from the viewpoint of measurement speed.

### 3.2. Design and Fabrication of the Sensor Device

**Figure 8:**
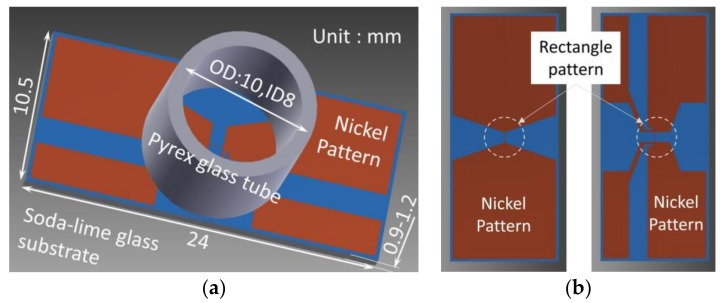
Design of the sensor device: (**a**) Overview and (**b**) nickel patterns for the 2-terminal-type (left) and 4-terminal-type (right).

The sensor device (Figure 8a) was fabricated as follows. A soda-lime glass substrate (S7213, thickness 0.9–1.2 mm; Matsunami Glass Industry., Ltd., Osaka, Japan) was cleaned ultrasonically in pure water and then methanol. The nickel thin film was sputtered on the substrate by the RF magnetron sputtering method (CFS-4ES-SS, Shibaura Mechatronics Corporation, Tokyo, Japan) at two thicknesses (25 and 75 nm). Nickel was selected because of its high temperature coefficient of resistance, its ease of etching, and its compatibility to the glass substrate. The adhesion of the nickel film was good as no delamination was observed after the simplified peeling test and even direct soldering could be performed. The pattern was formed by the standard photolithography technique. The positive-type photo resist (OFPR-800LB; Tokyo Ohka Kogyo Co., Ltd.; Kawasaki, Japan) was applied by the spin coating method, and the pattern was exposed by maskless exposure (Nano System Solutions, NanoSystem Solutions, Inc., Okinawa, Japan) with the minimum resolution of 1 µm. The resist was developed and the nickel film was etched by 10 wt % HCl aq. and 3 wt % HNO_3_ aq. The substrate was diced into the prescribed size (10.5 mm×24 mm) to complete the sensor chip fabrication.

**Figure 9:**
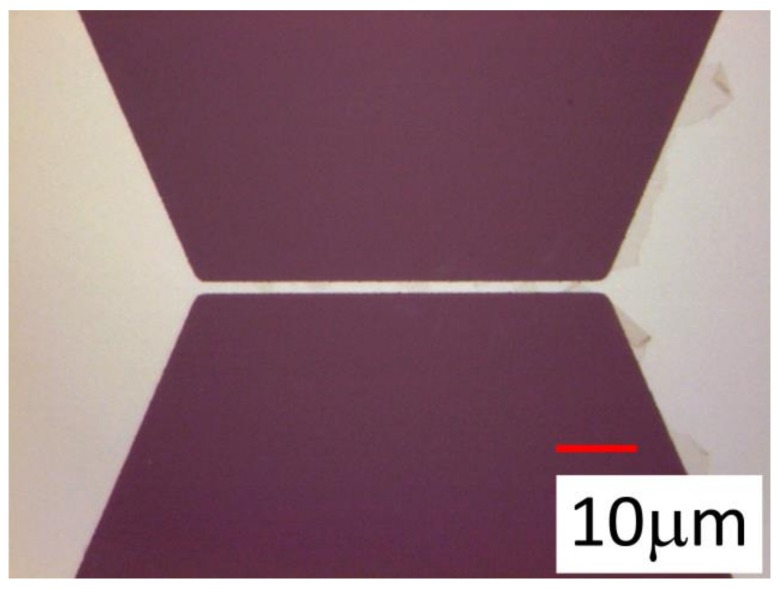
Photograph of the rectangle pattern on the sensor. The design was 2 µm wide and 50 µm long.

Two different types of nickel patterns were designed for the evaluation (Figure 8b). As we discuss later, the 4-wire configuration which divides the current input line and the voltage output line, was employed in our system. In the 2-terminal-type pattern (Figure 8b left), the separation of the current and the voltage line was not done on the sensor chip, but was done in the sensor wiring. In the 4-terminal-type pattern, the separation was done just neighboring the heat source rectangle area on the sensor chip. The various sizes of the rectangle pattern were designed as listed later. A photograph of the typical heat source rectangle area pattern on the sensor chip is shown in Figure 9.

After completion of the sensor chip patterning, a Pyrex^TM^ glass tube (inner diameter, 8 mm; outer diameter, 10 mm; length, approx. 20 mm, Ookabe Glass HD., Fukui, Japan) was glued to the chip surface using epoxy adhesive (Araldite Standard; Huntsman, TX, USA) to form the liquid sample container covering the heat source rectangle area as shown in Figure 8a. The adhesion quality was sufficiently good that no visible degradation was observed after more than 3 weeks of continuous usage with water sample and no serious problems occurred after more than 1 day of continuous usage with methanol sample.

**Figure 10:**
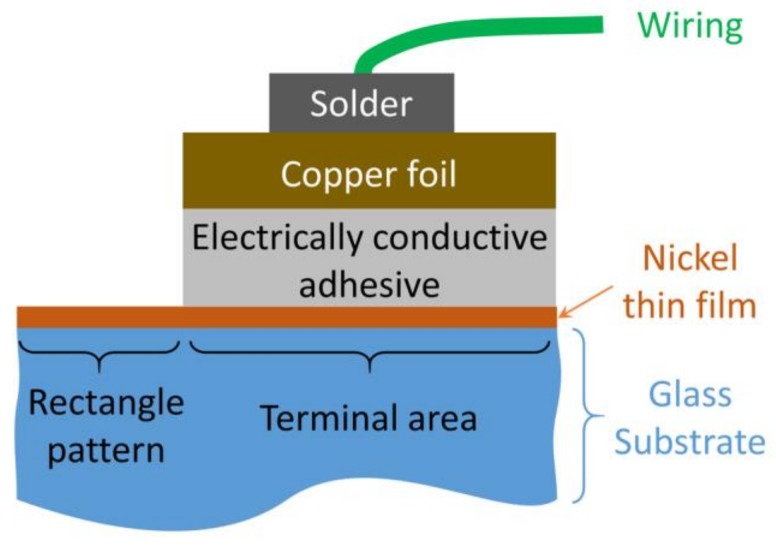
Wiring connection to the device terminal.

After completion of the sensor device fabrication, the electrical wiring was performed onto the terminal part of the device. Although direct soldering onto the nickel pattern was possible, the electrically conductive adhesive was used to connect the wiring and the chip terminal as depicted in Figure 10. An 18 µm thick copper foil was adhered on the terminal pattern and the wiring was soldered onto the foil. This terminal structure can minimize the parasitic resistance at the terminal pattern because of the very thin nickel film.

### 3.3. Detection Circuit

**Figure 11:**
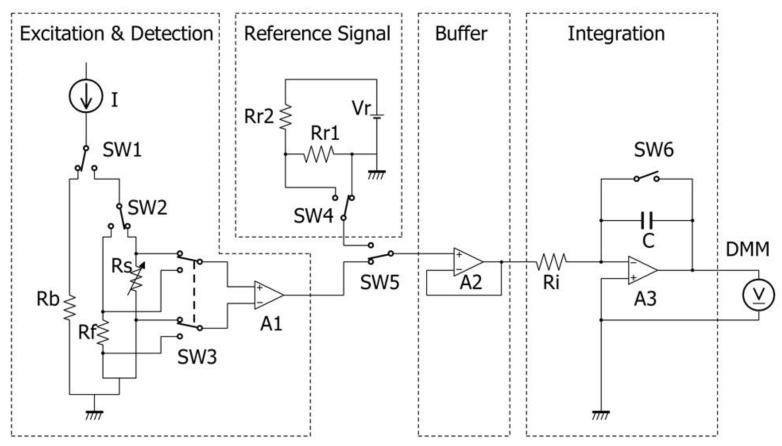
Schematic of the circuit diagram.

The schematic diagram of the detection circuit is shown in Figure 11. The ballast resistor Rb which had similar resistance to that of the sensor Rs, was connected in parallel with Rs, and the constant current source I was connected through the fast switch SW1 (TS5A3154; Texas Instruments, Dallas, TX, USA). A home-made current source was used because of the requirements for precision and response speed. A pulse signal with width of 0.4–10 µs was generated by the 10 MHz original oscillation crystal oscillator whose frequency tolerance was $\pm 15\;\times \;10^{-6}$ and the accumulated jitter was a few ps order; this pulse signal width could be used to define the heating time precisely.

The voltage drop at Rs was differentially detected by the instrumentation amplifier A1 (AD8421BR; Analog Devices, Norwood, MA, USA) whose gain stability at unity gain was 0.1 ppm/K, small signal bandwidth was 10 MHz, and settling time (0.001%) was 1 µs. The reference resistor Rf which also had a similar resistance to that of Rs and 0±5 ppm/K stability, was connected in parallel with Rs through the selector SW2. The zero TCR of Rf makes it possible to not only evaluate the stability of the current source, but also eliminate the noise influence intruding from the sensor wiring. The output of A1 was input to the buffer amplifier A2 (ADA4891-1; Analog Devices) through the selector SW5. SW5 was able to select the excitation and detection block and the reference signal block. In the reference signal block, the internal reference signal was made from the voltage reference (ADR441BRZ; Analog Devices), the voltage dividing circuit constructed by the precise resistor (0±5 ppm/K), and the fast switch SW4 controlled by the same frequency source of SW1. The reference signal block makes it possible to evaluate the stability of the subsequent stage of A2 and to cancel out its fluctuation.

The output of A2 was introduced to the integration block and the output of the integration was connected to the digital multi meter (AD7461A; DMM; Advantest, Tokyo, Japan). Our theoretical Equation (13) expresses the time-temperature response against the pulse heating. In order to digitize the identical signal experimentally, the analogue-digital conversion must be executed faster than the heating pulse. For our purpose, the required A/D frequency would be estimated as more than 10 MHz with resolution of more than 20 bits and these are difficult to realize using existing A/D converters. The integration block works like a peak hold circuit and its output can be digitized, although slowly by a highly precise A/D converter. By measuring the integral for different pulse widths, the original time–temperature response can be easily restored by taking its differential.

**Figure 12:**
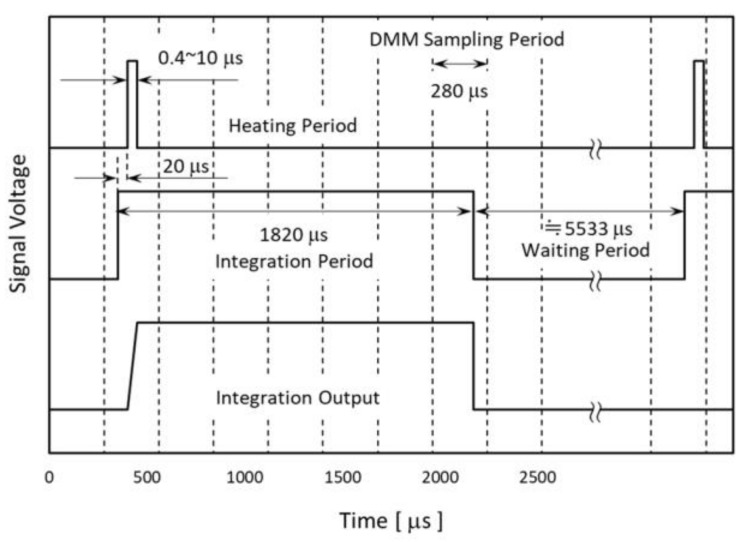
Timing chart of the detection circuit.

The integration period was defined by the fast switch SW6. The switch was controlled synchronously with SW1. In this paper, we set the integral period at 1820 µs and the delay time of the heating pulse at 20 µs just after the rise of the integral pulse. The timing chart is shown in Figure 12.

The data stored in the DMM were transferred to the PC. There were abnormal data which might be caused by the communication error with approximately 0.4% probability. Because the difference between the normal and the abnormal data could be clearly judged, the abnormal data were mechanically deleted from the data discussed in this paper.

### 3.4. Instrumentation

**Figure 13:**
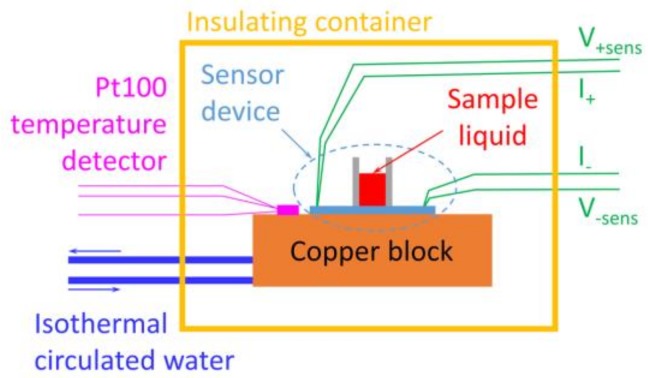
Schematic of the setup of the sensor device.

The sensor device was placed on a copper block (P-100S; 60 × 60 × 10 mm, Takagi Seisakusyo Co., Ltd, Kyoto, Japan) whose temperature was controlled by a circulating thermostat. A Pt100 temperature detector (FK 222-100-A Class A; Heraeus, Hanau, Germany) was also set on the copper block by means of the 3-wire configuration and it was used to monitor the block temperature in 0.01 K resolution and to control the temperature of the circulating water (E5CN-H; Omron, Kyoto, Japan). During the stable temperature period, the fluctuation of the temperature reading was within ±0.02 K. A schematic view of the setup is shown in Figure 13. Although it is not described in Figure 13, the resistor Rf and its wirings were also introduced just as in the sensor device.

### 3.5. Measurement Sample

Measurement samples were pure water which was purified by a water purification system (Direct-Q UV3; Merck, Darmstadt, Germany) and 99.5% conc. methanol purchased from Kishida Chemical Co., Ltd. (Osaka, Japan) (010-48665).

## 4. Results and Discussion

### 4.1. Evaluation Results of the Detection Circuit Stability

**Figure 14:**
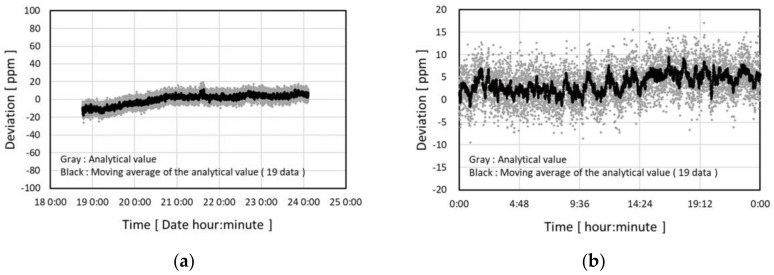
Estimation results of the long-term stability of the detection circuit: (**a**) 5 days; (**b**) 24 h.

The evaluation results of the long-term stability of the detection circuit using the reference signal block is shown in Figure 14. All the experiments below were done under conditions of 5 mA pulse current and 136 Hz pulse frequency. Pulse width of 10 µs was used in the experiment of Figure 14.

The circuitry was placed in an ordinary air-conditioned laboratory room and the room temperature fluctuated within 23±1 °C. Figure 14a shows stability during 5 days and Figure 14b shows the fluctuation within 24 h. The relative deviation from the average of the output during the whole evaluated period is taken as the vertical axes.

The data acquisition and the statistical processing for the data which was consistently applied in this paper is as described below. The voltage signal output from the integration block was analogue–digital converted and stored by the DMM as the sequence of voltage signal data of one 2.8 s period with a 280 µs interval (totally 10,000 data in one sequence). The sequence of the voltage signal data was transferred to a PC with the interval time of approximately 14 s. One sequence contained about 381 pulses. The voltage of each pulse was extracted from the sequence by a conventional signal processing method. The average of the pulse voltages from one sequence is called the analytical value. The gray points in Figure 14 show the relative temporal transition of the analytical value.

In Figure 14, the moving averages of 19 points of the analytical value are also plotted as black points for the purpose of stability analysis. In comparison with the gray points, the drift type noise dominates the fluctuation of the black points. The random noises are effectively reduced by taking the average of a total of 7239 pulses. The standard deviation of the residual fluctuation in the black points is 2.0 ppm and the difference between the maximum and minimum value is 11.1 ppm. They are equivalent to 0.0007 K and 0.004 K respectively, according to the sensor TCR value discussed below.

### 4.2. Evaluation Results of the Sensor Characteristics

The design parameter and the estimated static characteristics of nine sensor devices are listed in Table 1. No degradation of the nickel pattern was confirmed during the experiments.

#### 4.2.1. Electrical Resistance of the Rectangular Patterns

The electrical resistances of the rectangular patterns were evaluated as described below. The sample fluid was 400 µL of pure water. In order to avoid sample evaporation, the open end of the glass tube on the sensor device was sealed with a plastic paraffin film (Parafilm M; Bemis Company, Inc., Minneapolis, MN 55418, USA). The same sample volume and sealing method were applied to all the experiments in this paper. The temperature of the copper block was set at 27.5 °C and there was a waiting period to achieve the stable value. After the temperature stabilization, 5 or 10 µs heating pulses were applied to obtain 13 analytical values within a 10-min measurement. The average of the 13 analytical values was treated as one adopted value. During the 10 min, identical measurements were done for both the sensor Rs and the reference resistor Rf. The same measurements were executed three times and the averages of the three adopted values were calculated for both Rs and Rf. The reference resistor Rf was a highly precise metal foil resistor (FLAX100R00A; Alpha Electronics, Tokyo, Japan) and its resistance was 100 Ω±0.5% and the stability was 0±5 ppm/K. The resistance of the sensor device was calculated from the ratio of the average of the adopted values of Rs and Rf. The calculated resistance and estimated resistivity values are listed in Table 1. The actual measurement values (VK-9510, Keyence Corporation, Osaka, Japan) of the rectangle width were used to calculate the resistivity in order to consider the side etching.

**Figure 15:**
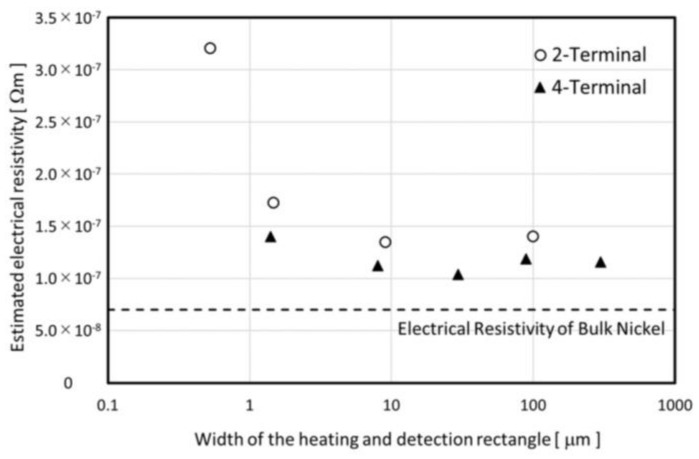
Estimated electrical resistivity as functions of the width of the rectangle pattern.

The resistivity of bulk nickel is about $7\times 10^{-8}\;\Omega m$ [27]. The resistivity of sensor #2 is 4.6 times larger than the bulk value, but no abnormality was confirmed from the simple estimation of the film resistance before the patterning process. The relationship between the width of the rectangle pattern and the resistivity is shown in Figure 15. The resistivity does not show any dependence on film deposition batch and film thickness, and the resistivity of less than 2 µm width patterns show clearly higher values. The 4-terminal-type pattern shows smaller resistivity than the 2-terminal-type pattern, even for the same pattern width condition. From these findings, the resistance values for less than 2 µm width patterns are supposed to contain not only the rectangle region but also the terminal part resistance.

More than 60% of the resistance of sensor #2 is estimated as the terminal part resistance. Sensor #2 also shows abnormal results in Section 4.2.3 and we discuss that later.

#### 4.2.2. Temperature Coefficient of the Sensor Resistance

**Figure 16:**
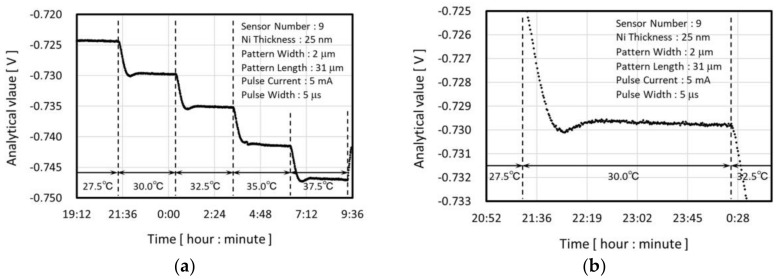
Example of the analytical value during the TCR evaluation (**a**) 1 cycle; (**b**) 30 °C setting period.

**Figure 17:**
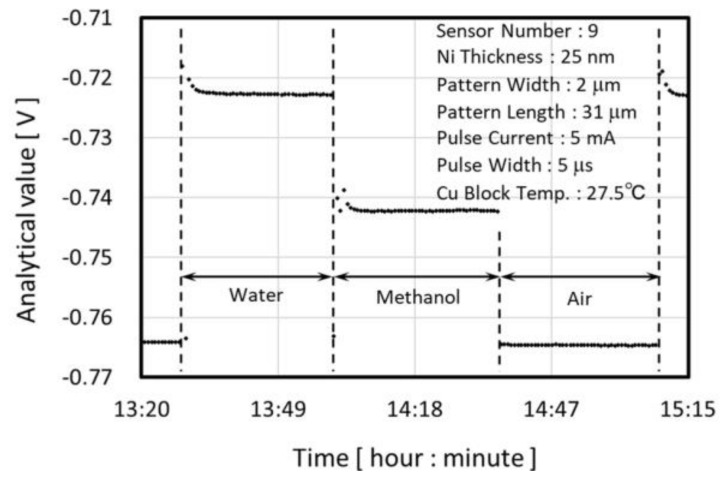
Example of the analytical value during the detection performance evaluation.

The TCR of the sensor devices was estimated as described below. Pure water was set as the sample, and the temperature of the copper block was changed from 27.5 to 37.5 °C in a step of 2.5 °C at regular 3-h intervals. An example of the temporal transition of the analytical value is shown in Figure 16. The undershoot which can be confirmed in Figure 16, is because of the response time of the temperature controller. As we can see later in Figure 17, the response of the sensor device is sufficiently fast. The average of approximately 20 analytical values (the adopted value) which were taken from the time period of 15 min after stabilization of the sensor output was used to calculate the sensor TCR.

The gradient of the linear approximation of 5 adopted values for 27.5–37.5 °C was divided by the adopted value for 32.5 °C and regarded as the estimated TCR value. The linearity of the adopted value against the temperature is good and the coefficient of determination exceeds $R^{2}=0.999$ for all of the sensor devices. The estimated value of TCR are listed in Table 1.

In contrast to the resistivity, the TCR does not show any dependence on the pattern design parameter. The TCR values are about 40% of the bulk nickel TCR [27]. Many research studies have been done regarding the difference of the electrical properties between bulk material and thin film [31]. In general, the TCR of the metal film tends to decrease as its thickness decreases to less than a particular thickness which is defined by the material, morphology, or other factors. The mean free path of the conduction electrons in nickel has been estimated to be around 20 nm. This means our devices experience some influence from the thickness. Our estimated TCR values agree well with previously reported values [32,33,34,35].

#### 4.2.3. Detection of the Thermal Property Difference

The performance of the detection of thermal property difference was evaluated. An example of the temporal transition of the analytical value during the evaluation is shown in Figure 17.

The temperature of the copper block was set at 27.5 °C and there was a waiting period to achieve stable temperature condition. After the temperature stabilization, about 13 analytical values were measured for 5 or 10 µs pulse widths within 10 min. The average of these analytical values was calculated as the adopted value. Using the identical measurement condition, we measured water, methanol and air. By regarding the whole measurement protocol as one cycle, three cycle measurements were executed and the average of the adopted values for each cycle were calculated and are called the evaluation value (E.V.).

The literature values of the thermal properties of the material used in our experiments are listed in Table 2.

The detection performance was evaluated using the index described below.

(22) $$\mathrm{Detection}\;\mathrm{index}=\;\left({E.V.}_{\mathrm{water}}-{E.V.}_{\mathrm{sample}}\right)/{E.V.}_{\mathrm{water}},$$

**Figure 18:**
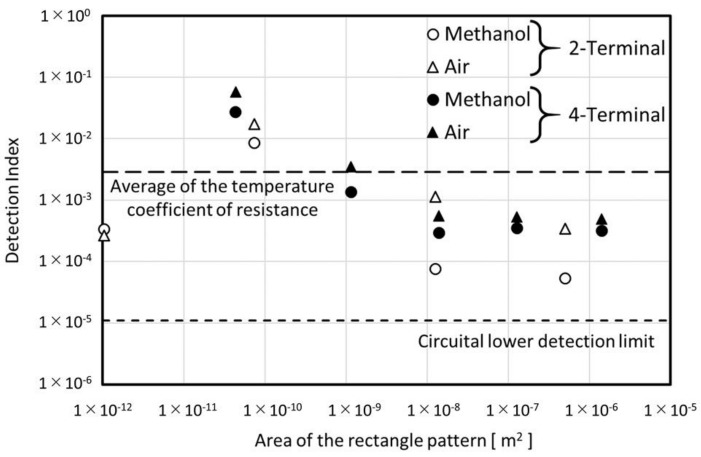
Comparison of the detection index for different rectangle areas.

The evaluated detection index is shown in Figure 18.

Although the TCR shows no dependence of the rectangle dimensions, the detection index has a tendency to increase as the rectangle area decreases. In the TCR estimation, the injected heating energy was canceled out by dividing the specific adopted value, and the temperature elevation induced by the heating pulse did not have any effect by considering the gradient of the linear approximation. In the detection index estimation, although the injected heating energy was also canceled out by dividing by the specific evaluation value, the difference of the temperature elevation induced by the heating between different samples was extracted by taking the difference of the evaluation value of the two fluids.

As we can see from Equation (13), the temperature elevation at the specific time t is defined by $p\;\left(\mathrm{unit}:\;J/\left(m^{2}\times s\right)\right)\;$ and the sample thermal properties. In the detection index estimation, the total injected heat $\left(1.2\times 10^{-8}-3.5\times 10^{-8}\;J\;\right)$ and the heating time (5, 10 μs) were almost constant in contrast to the rectangle area $\left(1.0\times 10^{-12}-1.4\times 10^{-6}\;m^{2}\right)$. Then the decreasing tendency in Figure 18 can be regarded as the dependence of the heat density on the detection index.

Above $1\times 10^{-8}\;m^{2}$, the area dependence disappears. This indicates the lower limitation of the temperature elevation detection. The limit seems ten times higher than the lower detection limit of the electrical circuit that was estimated in Section 4.1. The detection index of sensor #9 for methanol is $2.7\times 10^{-2}$. According to the thermal conductivity of water and methanol, the system limit of resolution can be estimated as about ∆0.0015 W/(m×K).

The detection index for sensor #2 shows an abnormal result. There are two reasons for this sensor behavior. The first is the influence of the resistance in the terminal pattern as already discussed in Section 4.2.1. In the discussion for Figure 18, all of the heat was assumed to be injected into the rectangle region. But from the discussion in Section 4.2.1, less than 40% of the heat was injected in the sensor #2 experiment. In addition the ratio of the resistance of the rectangle region was 40% of the total resistance of the sensor. Then the total sensitivity was reduced less than 16% compared to the situation for other sensors.

The second reason relates to the dimensions of the rectangle in sensor #2 itself. The non-negligible fraction of the injected heat to the rectangle region diffused from the detection area compared to the other sensors. As we noted in the principle section, the dimensionless parameter A is important for thinking about the heat diffusion:

(23) $$A=\frac{r}{\sqrt{4\alpha t}},$$

where r is the distance from the heat source, t is the heating time, and α is the thermal diffusivity of the media. The solution of the heat conduction equation can be expressed as the temporal and spatial integral of the exponential function which has $-A^{2}$ inside the function. When we think about the situation A=1, $\alpha \approx 5\;\times \;10^{-7}\;m^{2}/s$ (soda-lime glass), $t=10\times 10^{-6}\;s$, r is estimated as about $4\;\times \;10^{-6}\;m$. Sensor #2 was the only one for which both width and length were less than $4\;\times \;10^{-6}\;m$. In other words, for sensor #2, $f^{*}$ in Equation (13) was farther from 1 compared to the other sensors.

Figure 18 also shows the difference of the detection index between the methanol and the air samples. The ratios of the methanol to the air samples were around 0.5. These results agreed with results of our analytical model. If we use the existing free-standing model, these values should be 0.04 or less which are dominantly defined by the difference of the thermal properties between the methanol and the air samples.

In order to discuss further the validation of the analytical model using the experimental results, a precise calibration method must be established. Because our model includes information for the substrate material, the thermal properties of the substrate must be precisely evaluated. It is well known that the thermal properties of a solid are affected by many factors including density, presence of defects, and presence of impurities. As a consequence, they have relatively large uncertainties. We think every sensor device must be calibrated using a number of standard liquid samples which have certified thermal property values. It is not difficult to measure liquid sample thermal properties with an accuracy of better than 0.1% by using existing instruments. Details of a calibration method are a topic to be discussed in our subsequent paper.

#### 4.2.4. Discussion on the Existence of Thermal Contact Resistance

Our model does not consider the existence of thermal contact resistance as we mentioned in Section 2 Principle. The reported values of the thermal contact resistance between nickel thin film and silicon dioxide are around $10^{-9}-10^{-8}\;\left(m^{2}K\right)/W$ [39]. We had assumed the value of $5\;\times \;10^{-8}\;\left(m^{2}K\right)/W$ and calculated the temperature jump for the experimental conditions of sensors #3, #5, and #9. As a result, the influence was estimated to be around 4–8% of the total temperature increase. Intensive experimental evaluations are required for any further discussion if an application requires much better accuracy.

## 5. Conclusions

We have proposed the new analytical model for the MEMS-based non-steady thermal sensor for single-sided measurement. Our model is theoretically valid for a sensor length of less than 1 µm; this compares to existing models which are valid for a length of more than 1 mm under practical measurement conditions. The infinite value of the heat source length made our model converge to the existing THS analytical solution. The single-sided measurement makes it easy to measure high viscosity samples or non-fluid samples like living cells.

In order to realize sample miniaturization for around 1 µm scale, fast and precise electrical detection is required. We have also proposed simple electrical circuit architecture and a sensor design and confirmed their performance experimentally. The differences for sample thermal properties were successfully detected. The lower limit of the system resolution was estimated as about ∆0.0015 W/(m×K).

We theoretically estimated a 4–8% influence by the thermal contact resistance. Further experimental work is required to clarify the influence of the thermal contact resistance and to establish the precise calibration method.

## Figures and Tables

**Table 1:** List of the sensor design parameters and properties.

Sensor ID Number	Film Deposition Batch Number	Thickness of the Ni Thin Film	Pattern Information			Estimated Results		
			Terminal Type	Rectangle Pattern		Electrical Resistance	Electrical Resistivity	Temperature Coefficient of Resistance
				Width	Length			
				Design (Measured)	Design			
		nm		µm		Ω	Ω×m	ppm/K
#1	1	75	2	10 (9.1)	1400	277.8	1.44 × 10^−7^	2910
#2	2	25		1 (0.5)	2	48.9	3.2 × 10^−7^	2790
#3	3	75		2 (1.5)	50	78.2	1.7 × 10^−7^	2840
#4	4			100 (100.1)	5000	93.3	1.4 × 10^−7^	2830
#5	5	25	4	9 (8.0)	143	79.9	1.1 × 10^−7^	2880
#6				30 (29.4)	475	67.0	1.0 × 10^−7^	3020
#7				90 (89.2)	1425	75.8	1.2 × 10^−7^	2930
#8				300 (298.9)	4750	73.7	1.2 × 10^−7^	2870
#9	6			2 (1.4)	31	123.6	1.4 × 10^−7^	3100

**Table 2:** Literature values of the thermal properties of the materials used in our experiments.

	Properties	Density	Constant Pressure Specific Heat Capacity	Thermal Conductivity	Thermal Diffusivity ^2^
Material		kg/m^2^	kJ/(kg × K)	W/(m × K)	m^2^/s
Water ^1^		996	4.18	0.611	1.47× 10^−7^
Methanol ^1^		784	2.54	0.202	1.01 × 10^−7^
Air ^1^		1.16	1.01	0.0262	2.25 × 10^−5^
Nickel ^1^		8900	0.45	90	2.3 × 10^−5^
Soda-lime glass [36,37]		2500	0.8	1.0	5.0 × 10^−7^

^1^ The values were calculated as properties at 27.5 °C by using the values of [38]. The significant figures of the original values in [38] were 4 digits except for the thermal conductivity of methanol and the heat capacity and the thermal conductivity of nickel (3 digits). The accuracy of the values listed here was roughly estimated to be within 5%. ^2^ The values were calculated from the values of density, constant pressure specific heat capacity, and thermal conductivity.

## References

[1] Ritossa F. (1962). A new puffing pattern induced by temperature shock and DNP in Drosophila. Experientia.

[2] Tissieres A., Mitchell H.K., Tracy U.M. (1974). Protein synthesis in salivary glands of Drosophila melanogaster: Relation to chromosome puffs. J. Mol. Biol..

[3] Hartl F.U., Bracher A., Hayer-Hartl M. (2011). Molecular chaperones in protein folding and proteostasis. Nature.

[4] Magrane J., Smith R.C., Walsh K., Querfurth H.W. (2004). Heat shock protein 70 participates in the neuroprotective response to intracellularly expressed β-Amyloid in neurons. J. Neurosci..

[5] Caterina M.J., Schumacher M.A., Tominaga M., Rosen T.A., Levine J.D., Julius D. (1997). The capsaicin receptor: A heat-activated ion channel in the pain pathway. Nature.

[6] Tominaga M., Caterina M.J. (2004). Thermosensation and pain. J. Neurobiol..

[7] Takayama Y., Furue H., Tominaga M. (2017). 4-isopropylcyclohexanol has potential analgesic effects through the inhibition of anoctamin 1, TRPV1 and TRPA1 channel activities. Sci. Rep..

[8] McLaughlin E., Tye R.P. (1969). Thermal Conductivity 2.

[9] Assael M.J., Antoniadis K.D., Wakeham W.A. (2010). Historical Evolution of the Transient Hot-Wire Technique. Int. J. Thermophys..

[10] Wang H., Sen M. (2009). Analysis of the 3-omega method for thermal conductivity measurement. Int. J. Heat Mass Tran..

[11] Moskal D., Martan J., Lang V., Švantner M., Skála J., Tesař J. (2016). Theory and verification of a method for parameter-free laser-flash diffusivity measurement of a single-side object. Int. J. Heat Mass Tran..

[12] Beigelbeck R., Nachtnebel H., Kohl F., Jakoby B. (2011). A novel measurement method for the thermal properties of liquids by utilizing a bridge-based micromachined sensor. Meas. Sci. Technol..

[13] Mahdavifar A., Navaei M., Hesketh P.J., Findlay M., Stetter J.R., Hunter G.W. (2015). Transient thermal response of micro-thermal conductivity detector (TCD) for the identification of gas mixtures: An ultra-fast and low power method. Microsyst. Nanoeng..

[14] Struk D., Shirke A., Mahdavifar A., Hesketh P.J., Stetter J.R. (2018). Investigating time-resolved response of micro thermal conductivity sensor under various modes of operation. Sens. Actuators B Chem..

[15] Van der held E.F.M., Van Drunen F.G. (1949). A method of measuring the thermal conductivity of liquids. Physica.

[16] Sommerfeld A. (1894). Zur analytischen Theorie der Wärmeleitung. Math. Ann..

[17] Bellman R., Marshak R.E., Wing G.M. (1949). Laplace transform solution of two-medium neutron ageing problem. Philos. Mag..

[18] Gustafsson S.E., Karawacki E. (1983). Transient hot-strip probe for measuring thermal-properties of insulating solids and liquids. Rev. Sci. Instrum..

[19] Birge N.O. (1986). Specific-heat spectroscopy of glycerol and propylene-glycol near the glass-transition. Phys. Rev. B.

[20] Takegoshi E., Imura S., Hirasawa Y., Takenaka T. (1982). A method of measuring the thermal-conductivity of solid materials by transient hot-wire method of comparison. Bull. JSME.

[21] Shendeleva M.L. (2001). Reflection and refraction of a transient temperature field at a plane interface using Cagniard-de Hoop approach. Phys. Rev. E.

[22] Shendeleva M.L. (2004). Instantaneous line heat source near a plane interface. J. Appl. Phys..

[23] Katayama T., Morishima K. Time evolution of the heat diffusion phenomenon from the point source near the interface. Int. J. Therm. Sci..

[24] Gustafsson S.E. (1967). A non-steady-state method of measuring thermal conductivity of transparent liquids. Zeitschrift für Naturforschung.

[25] Gustafsson S.E., Karawacki E., Khan M.N. (1979). Transient hot-strip method for simultaneously measuring thermal-conductivity and thermal-diffusivity of solids and fluids. J. Phys. D Appl. Phys..

[26] Carslaw H.S., Jaeger J.C. (1959). Conduction of Heat in Solids.

[27] National Astronomical Observatory (2007). Chronological Scientific Tables.

[28] Cahill D.G., Pohl R.O. (1987). Thermal-conductivity of amorphous solids above the plateau. Phys. Rev. B.

[29] Gustafsson S.E., Chohan M.A., Ahmed K., Maqsood A. (1984). Thermal properties of thin insulating layers using pulse transient hot strip measurements. J. Appl. Phys..

[30] International Organization for Standardization ISO 5725-1:1994(en) Accuracy (Trueness and Precision) of Measurement Methods and Results—Part 1: General Principles and Definitions. https://www.iso.org/obp/ui/#iso:std:iso:5725:-1:ed-1:v1:en.

[31] Dinh T., Phan H.P., Qamar A., Woodfield P., Nguyen N.T., Dao D.V. (2017). Thermoresistive effect for advanced thermal sensors: Fundamentals, design considerations, and applications. J. Microelectromech. Syst..

[32] Belser R.B., Hicklin W.H. (1959). Temperature coefficients of resistance of metallic films in the temperature range 25° to 600 °C. J. Appl. Phys..

[33] Ghosh C.K., Pal A.K. (1980). Electrical resistivity and galvanomagnetic properties of evaporated nickel films. J. Appl. Phys..

[34] Starý V., Šefčik K. (1981). Electrical resistivity and structure of thin nickel films-effect of annealing. Vacuum.

[35] Angadi M.A., Udachan L.A. (1981). Electrical properties of thin nickel films. Thin Solid Films.

[36] (2016). Asahi Glass Architectural Glass General Catalog. https://www.asahiglassplaza.net/catalogue/sougou_gijutsu/0004a.pdf.

[37] (2015). Nippon Sheet Glass Architectural Glass General Catalog—Technical Reference. http://glass-catalog.jp/pdf/g32-010.pdf.

[38] The Japan Society of Mechanical Engineers (2009). JSME Data Book: Heat Transfer.

[39] Chien H.C., Yao D.J., Hsu C.T. (2008). Measurement and evaluation of the interfacial thermal resistance between a metal and a dielectric. Appl. Phys. Lett..

